# From the Cajal alumni Achúcarro and Río-Hortega to the rediscovery of never-resting microglia

**DOI:** 10.3389/fnana.2015.00045

**Published:** 2015-04-14

**Authors:** Marie-Ève Tremblay, Cynthia Lecours, Louis Samson, Víctor Sánchez-Zafra, Amanda Sierra

**Affiliations:** ^1^Centre de Recherche du CHU de Québec, Axe NeurosciencesQuébec, QC, Canada; ^2^Département de médecine moléculaire, Université LavalQuébec, QC, Canada; ^3^Achúcarro Basque Center for Neuroscience, Bizkaia Science and Technology ParkZamudio, Spain; ^4^Department of Neurosciences, University of the Basque CountryLeioa, Spain; ^5^Ikerbasque FoundationBilbao, Spain

**Keywords:** microglia, discovery, Cajal, Achúcarro, Río-Hortega, imaging, neuroanatomy, phagocytosis

## Abstract

Under the guidance of Ramón y Cajal, a plethora of students flourished and began to apply his silver impregnation methods to study brain cells other than neurons: the neuroglia. In the first decades of the twentieth century, Nicolás Achúcarro was one of the first researchers to visualize the brain cells with phagocytic capacity that we know today as microglia. Later, his pupil Pío del Río-Hortega developed modifications of Achúcarro's methods and was able to specifically observe the fine morphological intricacies of microglia. These findings contradicted Cajal's own views on cells that he thought belonged to the same class as oligodendroglia (the so called “third element” of the nervous system), leading to a long-standing discussion. It was only in 1924 that Río-Hortega's observations prevailed worldwide, thus recognizing microglia as a unique cell type. This late landing in the Neuroscience arena still has repercussions in the twenty first century, as microglia remain one of the least understood cell populations of the healthy brain. For decades, microglia in normal, physiological conditions in the adult brain were considered to be merely “resting,” and their contribution as “activated” cells to the neuroinflammatory response in pathological conditions mostly detrimental. It was not until microglia were imaged in real time in the intact brain using two-photon *in vivo* imaging that the extreme motility of their fine processes was revealed. These findings led to a conceptual revolution in the field: “resting” microglia are constantly surveying the brain parenchyma in normal physiological conditions. Today, following Cajal's school of thought, structural and functional investigations of microglial morphology, dynamics, and relationships with neurons and other glial cells are experiencing a renaissance and we stand at the brink of discovering new roles for these unique immune cells in the healthy brain, an essential step to understand their causal relationship to diseases.

## The discovery of microglia

To us current investigators of microglia it is difficult to appreciate the 100-year research that has led us to where we are today. This road was plagued with obstacles, from the development of novel methods to visualize cells, to the difficulties in comparing results from different labs, which hindered the reach of a consensus nomenclature for the different cell types that constitute the neuroglia of the central nervous system (CNS). Neuroglia was first described by Virchow in 1846 as an adhesive substance connecting neurons, but it took 75 more years to realize that the neuroglia is composed of cells belonging to three major types: astrocytes, oligodendrocytes, and microglia (Garcia-Marin et al., 2007). One particularly critical point in this discovery was the systematic testing of different methods of fixation and impregnation of the brain tissue to allow the selective and complete staining of the different types of neuroglial cell populations. Most of the impregnation methods relied on the use of silver ions combined with other metals to bind to different (unknown) proteins, many of which are still being used by neuropathologists. Among these, labeling methods were eventually found that allowed the complete visualization and identification of microglia in 1919. In this process, two Spanish researchers were instrumental: Nicolás Achúcarro and Pío del Río-Hortega, both alumni of the Santiago Ramón y Cajal School. In spite of their limited microscopy techniques, these researchers were thorough anatomists that from the simple observation of fixed tissue were able to infer important postulates about the nature (ectodermic vs. mesodermic origin), structural plasticity (motility), and function (phagocytosis of neuronal debris) of microglia that are still valid today (Kettenmann et al., 2011).

In the early 1900s, Cajal was the undisputable world leader in functional neuroanatomy. His critical contributions to our understanding of the CNS granted him, along with the prominent Italian neuroanatomist Camilo Golgi, a 1906 Nobel prize in Medicine. Cajal's Laboratory of Biological Research was located in Madrid, Spain, where he trained many generations of neuroanatomists in the development of new methods, systematic observation of the brain tissue, and detailed drawing to provide functional hypotheses about the roles played by the different types of brain cells. Cajal is best known for his seminal observations of neurons and their connectivity using the Golgi staining, leading to propose in 1888 the “neuron doctrine” (as opposed to the “reticular theory”), but by the end of the century he became very interested in neuroglia and started to develop novel methods to visualize them. In 1897 he published his first paper entirely devoted to neuroglia, where he speculated that a main role of glial cells was to preserve neuronal circuits and avoid inappropriate contacts (Ramón y Cajal, 1897; Garcia-Segura, 2002; Navarrete and Araque, 2014). At that time, the term “astrocyte” had already been introduced by Michael von Lenhossék (Garcia-Marin et al., 2007) and used to describe both protoplasmic and fibrous star-shaped cells. It was also clear that astrocytes were not the only type of neuroglial cell in the brain. In fact, a myriad of cells had already been described by different investigators each using their own methods of staining, but considering the lack of documentation available today, it still remains unclear whether they were actually discussing about the same cell types (Table 1).

**Table 1:** **The first steps of glia research**.

**Cell type**	**Year**	**Author**	**Term**	**Origin**	**Function**	**First location**
Glia	1856	Virchow	Neuroglia	Ectodermal	Connection with neurons	Brain tissue
Astrocyte	1891	Van Lenhossék	Astrocyte	Ectodermal	Structural support	Brain tissue
Microglia and oligodendroglia	1913	Cajal	Third element	Mesodermal		Brain tissue
Microglia	1841	Gluge	Inflammatory corpuscles	Mesodermal	Phagocytosis	Damaged brain
Microglia	1856	Virchow	Foam cells	Mesodermal	Phagocytosis	Atherosclerosis plaques
Microglia	1899	Nissl	Rod cells	Mesodermal	Phagocytosis	Cerebral palsy
Microglia	1908	Achúcarro	Granuloadipose cells	Ectodermal/ Mesodermal	Phagocytosis	Brain of rabbits with rabies
Microglia	1910	Merzbacher	Scavenger cells	Mesodermal	Phagocytosis	Demyelinating CNS disorder
Microglia	1919	Del Río-Hortega	Microglia (mesoglia)	Mesodermal	Phagocytosis	Brain tissue
Oligodendroglia	1899	Robertson	Mesoglia	Mesodermal	Phagocytosis	Human and canine brain
Oligodendroglia	1921	Del Río-Hortega	Oligodendroglia (interfascicular glia)	Ectodermal	Support, isolation, nutrition	Brain tissue

*Table of the different terms used by the listed authors to designate glial cells, and their opinion on their presumed origin and function. Thorough accounts on the history of their discoveries can be found elsewhere (Rezaie and Hanisch, 2014). After Virchow coined the term “neuroglia” in the mid-nineteenth century to describe a substance that connected neurons, different researchers observed what seemed to be different types of glial cells in a variety of pathological conditions. Astrocytes (protoplasmic and fibrous) were readily identified by Van Lenhossék, but the rest of the neuroglial cell types received different names under different researchers and it was unclear whether they were in fact the same cell types. Ramón y Cajal grouped them under the term “third element,” but it was Río-Hortega who finally settled the issue and unambiguously discriminated between oligodendrocytes and microglia*.

To the Cajal School belongs the Basque-born psychiatrist Nicolás Achúcarro, both a colleague and an alumnus of Cajal (Vitoria Ortiz, 1977). After graduating as a medical doctor at the University of Madrid, Spain in 1904, Achúcarro traveled to Munich, Germany, to work in Alois Alzheimer's lab as a scientist and psychiatrist. There he became interested in neuroglia, and particularly in the rod cells *(Stäbchenzellen*), a cell type that Franz Nissl had discovered in 1898 while observing human autopsy cases of mentally ill patients with paralysis (Vitoria Ortiz, 1977; Rezaie and Hanisch, 2014). Achúcarro was able to visualize these cells in the brains of rabbits infected with rabies or damaged by focal or inflammatory injury using a Scharlach Red (specific for fatty tissue) and hematoxylin (which stains nuclei) staining. In particular he observed cells whose shape was adapted to that of neurons, localized around the “necrotic foci” (*sic*) in the pyramidal cell layer of the hippocampus. These cells were full of fatty degeneration products (that were called “protagonoid substances”), likely resulting from the degeneration of nervous structures. Since these “granuloadipose” cells were concentrated around the lesion area, he hypothesized that their role was to phagocytose the damaged neurons. Further, he thought that their elongated, rod shape was related to their active movement between the degenerating neurons (Achúcarro, 1909; de Castro, 1977).

**Figure 1:**
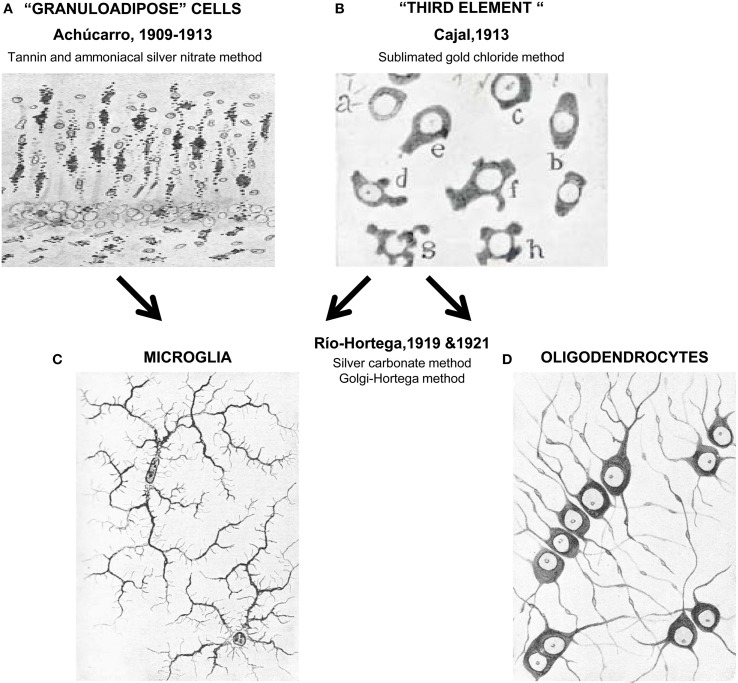
**Unraveling the nature of the “third element.”** In the early years of the twentieth century Achúcarro developed a novel method of staining (tannin and amoniacal silver nitrate) that allowed him to visualize what were at the time called “granuloadipose cells” that is, a group of cells that seemed to phagocytose degradation materials in the brain of rabid rabbits **(A)**. Stimulated by the interest of Achúcarro in glia, Cajal developed his famous sublimated gold chloride method that allowed him to beautifully visualize astrocytes but left the remaining neuroglial cells poorly stained (a–h, in **B**). To describe these apolar, dwarf cells he introduced the term “third element” of the nervous system. Río-Hortega continued the work of Achucarro and in 1919 developed his silver carbonate method, finally enabling him to resolve microglia **(C)** from what he initially called interfascicular cells and later oligodendrocytes (using his Golgi-Hortega method; **D**). Achúcarro's images are reprinted with permission from Achúcarro (1909). Cajal's image is reprinted with permission from Garcia-Marin et al. (2007). Río-Hortega's images are reprinted with permission from Río-Hortega (1919c).

After leading the Laboratory of Pathological Anatomy of the Federal Psychiatric Hospital in Washington DC, USA, Achúcarro returned to Madrid to join Cajal's laboratory. In 1910 he set up his own “Histology Laboratory” at the Students' Residency, which was part of the Free Teaching Institution, a precursor of the Spanish National Research Council. From Cajal he acquired the latest staining techniques and developed his own tannin and ammoniacal silver nitrate method (Achúcarro, 1911). With his staining, Achúcarro was able to clearly differentiate phagocytic, granuloadipose, and non-fibrous rod cells, from stellate cells with vascular end-feet and neuroglial fibrilles, which respectively correspond to our current understanding of microglia and astrocytes (Achúcarro, 1913) (Figure 1). Achúcarro strongly supported the idea that neuroglial cells must exert other functions than merely supporting neurons, an idea that had been widely accepted since Virchow. It was also clear to him that glial cell dysfunction could itself produce brain diseases, even without any primary damage to neurons (Achúcarro, 1913). This idea of glial cells primarily causing “gliopathies,” as opposed to secondarily reacting to neuronal damage in “neuropathies,” is re-emerging in today's research (Verkhratsky et al., 2012).

Another strong matter of discussion at the time concerned the origin of the granuloadipose cells. Achúcarro had initially observed what seemed to be an intermediate form between the granuloadipose cells and astrocytes, leading him to conclude that granuloadipose cells were derived from neuroglia, and thus had an ectodermic origin, supporting the thesis of many researchers including Nissl, who later reconsidered this theory (Achúcarro, 1913). Later, he observed what seemed to be granuloadipose cells migrating into the brain parenchyma from blood vessels, as Alzheimer had stated before, and inferred that some of these cells could simply be circulating monocytes (de Castro, 1977). However, there was no method available at that time to discriminate between these two alternative hypotheses. In fact, the neuroectodermic origin of astrocytes and the bone-marrow origin of circulating monocytes were found relatively early, but the unique origin of microglia as cells derived from the embryonic yolk sac had remained unknown (Alliot et al., 1999) and was only recently confirmed using fate mapping strategies (Schulz et al., 2012; Ginhoux et al., 2013; Kierdorf et al., 2013).

The work of Achúcarro on granuloadipose cells sparked the interest of Cajal on these cells, although they both acknowledged that the tannin and ammoniacal silver nitrate method used by Achúcarro was producing a partial and inconsistent labeling (de Castro, 1977; Vitoria Ortiz, 1977). This led Cajal to recall his former observations when he solely used formol as a fixative, to obtain a more selective staining of neuroglial processes, and to ultimately develop his formol uranium nitrate and sublimated gold chloride method (Ramón y Cajal, 1913; de Castro, 1977; Vitoria Ortiz, 1977), which soon became the standard to visualize fibrous and protoplasmic neuroglia (i.e., astrocytes) (Garcia-Marin et al., 2007). Nonetheless, this method barely stained the other type(s) of glial cell(s), and in 1913, Cajal introduced the controversial term “third element” to describe what he thought was the remaining class of glial cells: dwarf, adendritic, and apolar cells of the white matter, with perineuronal and perivascular location, and a mesodermal origin (Ramón y Cajal, 1913; Garcia-Marin et al., 2007).

These studies could not be pursued as Achúcarro became gravely ill in 1915 and passed away at a young age in 1918 from a self-diagnosed Hodgkin's lymphoma. He was deeply missed by his colleagues and Cajal, who wrote a wholehearted obituary (Ramón y Cajal, 1977). Nonetheless, his laboratory remained active and a student of his, Pío del Río-Hortega, was designated by Cajal to take the lead (Cano Diaz, 1985). Río-Hortega graduated in Medicine at the University of Valladolid, Spain in 1905. After unsuccessfully trying to join Cajal's lab for unclear reasons, Río-Hortega worked as a clinician and completed his doctorate courses at the University of Madrid, to finally obtain his PhD title from the University of Valladolid for his work on brain tumors in 1908. In 1913 he returned to Madrid and was admitted to Achúcarro's lab, with whom he developed a close master-student relationship (Prados y Such, 1977; Cano Diaz, 1985). After some brief stays in London and Berlin, the First World War forced Río-Hortega to return to Achúcarro's lab in 1915, which was now located just next to Cajal's Laboratory of Biological Research (Prados y Such, 1977; Cano Diaz, 1985). Río-Hortega professed a great admiration for Achúcarro, whom he considered an ingenuous and stimulating, benevolent and generous mentor (Prados y Such, 1977).

Notwithstanding his great scientific contributions and large body of publications, Río-Hortega entered in strife with some members of the Cajal School and Cajal himself (Cano Diaz, 1985; Rezaie and Hanisch, 2014). It is unclear whether behind this conflict were purely scientific reasons or more personal problems (Cano Diaz, 1985). The fact is that Río-Hortega's findings contradicted Cajal's view of the “third element” and that he left Cajal's lab in 1919 to establish his own lab back at the Student's Residency in 1920 (Cano Diaz, 1985; Rezaie and Hanisch, 2014). Río-Hortega was particularly involved with the search for simple, specific, and replicable methods to stain the nervous tissue, because he acknowledged, like Cajal before him, that novel findings required novel techniques (Cano Diaz, 1985). He acquired the methods of Cajal and Achúcarro, of Golgi and others and developed many variations, among them his famous silver carbonate method (a modification of Achúcarro's ammoniacal silver method) which provided an exceptional visualization of glial cells (Río-Hortega, 1916, 1917). With this method, Río Hortega was able to obtain smaller micelles that impregnated the finest details of the brain tissue (Rezaie and Hanisch, 2014) and revealed in great depth the morphology of what he classified as two independent cell types: microglia and interstitial cells (which he later named oligodendrocytes) (Río-Hortega, 1919a,b,c). Río-Hortega also realized that these two cell types were in fact what Cajal had called “adendritic” cells and that the master had not been able to discriminate between them because of an incomplete visualization of their processes using the sublimated gold chloride method of staining.

To Río-Hortega, it was clear that microglia have a small, dark nucleus, no signs of centrosome, a Golgi apparatus, some gliosomes (granules), and a profuse tree of processes: *“[microglia] are characterized by their small, dark nucleus enveloped by scant protoplasm and its long, tortuous, ramified expansions adorned with lateral spines”* (Río-Hortega, 1919b). His drawings of these cells stained with the silver carbonate method shockingly resemble our current labeling of microglia using modern immunostaining and transgenic labeling strategies (Figure 1), revealing the strengths of his fixation and staining techniques, as much as the exceptional quality of his artistic talents. In addition, Río-Hortega was a skillful writer who provided a clear articulation of systematically collected evidences and arguments in support of his conclusions. His technical prowess led him to discover that microglia are regularly distributed throughout the brain, showing a higher density in gray than in white matter areas (Río-Hortega, 1919c). He also proposed that, in contrast to astrocytes, microglia are highly migratory and structurally dynamic, because their shape adapts to the contours of the other nervous structures (Río-Hortega, 1919b), as Achúcarro had observed before him (Achúcarro, 1909). In his own words: “*The plasticity of their protoplasm when they insinuate through the interstices that separate the fibers as they follow their cross directions, when they go parallel to the cells and wrap them in their processes, and when they stretch in places where the nerve structures follow the same direction, and develop their expansions in the same way as neuron processes form their plexuses, and so on, show very clearly that their shape is mutable and conditional; that their protoplasm is capable of plasticity; and that they have, in short, a quality inherent to the migrant corpuscles, among which, in all probability, microglia must be included.”* (Río-Hortega, 1919b).

But he believed that it was during pathological conditions that microglia showed their true nature: “[…] *the nomadic nature of this [microglia] is best revealed during the destructive processes of the nervous tissue, when the apparent rest that it enjoys during normality turns into migratory and phagocytic activity*” (Río-Hortega, 1919b). Following the time course of a stab wound in the newborn cat, he proposed that microglia transformed into the granuloadipose cells of Achúcarro, becoming hypertrophic or ameboid and full of lipidic phagocytic inclusions (Río-Hortega, 1919c). Río-Hortega described a full progressive-regressive (*sic*) morphological cycle of microglia, from the ramified, sedentary (*sic*) forms found in normal physiological conditions to the amoeboid, phagocytic forms observed during injury, which to him resembled the morphology they display during development: “[…] *the forms acquired by microglia while passing, in pathological cases, from the normal or sedentary form to the globular form, are an accurate reflection of those which are successively acquired over the course of normal development. Our latest research confirms that in their evolution microglia go from the rounded to the stellar shape with branches, while in their involution they tend to regain the original form*” (Río-Hortega, 1919c). This analysis of microglial function based on their morphology has permeated even to our days, and has for many years been interpreted as a cascade of stereotypical activation from the ramified, resting form to the “activated,” hypertrophic cell, culminating into the phagocytic amoeboid state (Graeber, 2010; Streit et al., 2010; Tremblay et al., 2011). As we will discuss later, even though all those morphological stages can be observed in particular contexts of health or disease, the cascade of microglial “activation” in itself is now viewed as an oversimplification of microglial functional repertoire.

Río-Hortega's experiments also settled the problem of the granuloadipose cells nature that had been concerning researchers since Nissl back in 1898, clearly establishing them as microglia for once. Nonetheless, the origin of microglia still remained undetermined as discussed above. To Cajal, the “third element” was composed solely of cells of mesodermic origin. Río-Hortega, however, argued that the problem dwelt in the confusion between different cell types (i.e., microglia and oligodendrocytes), thus leading to contradictory observations (Río-Hortega, 1919b). He stated that oligodendrocytes had an ectodermic origin, and were part of the neuroglia. As astrocytes, oligodendrocytes adopted a permanent shape and location once development was completed. They had a round or polyhedral, epithelial-like cell body; few, long and poorly ramified processes; and were preferentially located in the white matter, aligned with the nerve tracts (Río-Hortega, 1919b). He proposed that oligodendrocytes were homologous to the Schwann cells of the peripheral nervous system and formed membrane expansions around the myelin wraps, which at the time were thought to be of axonal origin, although to him the analogy between oligodendrocytes and Schwann cells rather evidenced a glial origin of myelin (Río-Hortega, 1922). These ideas were initially contested by alumni of Cajal such as Lorente de No and de Castro (1923). De Castro later changed his mind and fully acknowledged the relevance of Río-Hortega's discoveries (de Castro, 1977). In contrast to oligodendrocytes, Río-Hortega claimed that microglia were clearly of mesodermic origin because of their late appearance in the brain parenchyma during development, often in close association with blood vessels. Microglia also showed strong analogies of shape, staining properties, nuclear structure, and phagocytic capacity with circulating monocytes (Río-Hortega, 1919a). Considering these properties as strong evidence for an origin outside of the brain, he believed that microglia truly represented a “third element” (Cano Diaz, 1985; Gill and Binder, 2007). In fact, he profusely referred to microglia as “mesoglia” (Río-Hortega, 1919b).

Río-Hortega eventually published his findings in Cajal's journal (Rio-Hortega, 1920) but Cajal answered in the same issue (Ramón y Cajal, 1920) that “mesoglia” had already been described back in 1900 by a Scottish researcher, William Ford Robertson (Rezaie and Hanisch, 2014). Robertson had used a platinum method to visualize these “mesoglia,” which he described to be of mesodermal origin, not related to nerve fibers, and phagocytic due to their lipidic inclusions (Iglesias-Rozas, 2013). However, Robertson's paper did not provide any images and it seems that his staining was particularly difficult to reproduce (Iglesias-Rozas, 2013; Rezaie and Hanisch, 2014). Cajal acknowledged that his “third element” was in fact composed of three different cell types whose morphology, structure and function were likely different: dwarf satellites, the interfascicular cells of Río-Hortega, and the microglia or Robertson/del Río cells (Ramón y Cajal, 1920). To Cajal, the priority in the discovery of microglia was Robertson's, although he conceded that he had not been able to read Robertson's original work but rather citations by other authors (Ramón y Cajal, 1920). The conundrum was solved by Wilder Penfield, an American neurosurgeon who visited Rio-Hortega's lab to learn his techniques and apply them to his epilepsy research (Gill and Binder, 2007; Rezaie and Hanisch, 2014). Penfield was able to compare Robertson and Río-Hortega's preparations. His observations suggested that Robertson's “mesoglia” were in fact Río-Hortega's oligodendrocytes, which decisively prompted to abandon the term “mesoglia” altogether (Gill and Binder, 2007; Rezaie and Hanisch, 2014). Although Robertson was indeed the first researcher to visualize oligodendrocytes, he was notoriously wrong in his claims about their origin and function. It was Penfield indeed who in 1924 finally settled down the dispute and established that the three main types of glial cells in the brain are astrocytes, oligodendrocytes, and microglia, giving the full credit to Río-Hortega for his discovery of microglia and oligodendrocytes (Gill and Binder, 2007). The initial reluctance of Cajal and his alumni to accept Río-Hortega's findings tarnished his reputation in Spain, despite his worldwide recognition. Nonetheless, the long-expected reconciliation with Cajal finally took place in 1928, when the old master was 77 years old (Cano Diaz, 1985).

In the meantime, Río-Hortega continued to study microglia on his own, in his small laboratory at the Student's Residency. He was particularly interested in their phagocytic function, which he described thoroughly (Río-Hortega, 1919c). In particular, he found that microglia contained large amounts of granularities, some of which were enclosed in vacuoles, as early as they appeared in the nervous parenchyma during development. He hypothesized that “*before young neurons and embryonic neuroglia acquire their definitive shape and location, [may] occur the fragmentation of some delicate appendices or phenomena of disintegration and de-assimilation that require the intervention of microglial macrophages to gather and transform the resulting debris”* (Río-Hortega, 1919c). Further, in foci of experimental necrosis (*sic*) microglia appeared as voracious macrophages, filled by adipose granules, as well as entire erythrocytes and leukocytes. The need for an increased phagocytosis, argued Río-Hortega, fueled microglia to proliferate during pathological conditions, an interesting observation that deserves to be tested experimentally. Indeed, after migration and phagocytosis, proliferation was to Río-Hortega the ultimate response of microglia to injury. Microglial proliferation, reasoned Río-Hortega, explained why the damaged tissue was soon filled with microglia in the absence of infiltration from circulating monocytes (Río-Hortega, 1919c). Even today, the infiltration of these cells into the brain, and their differential contribution to brain disorders compared with resident microglia remains an open area of study (Gomez-Nicola and Perry, 2014; Prinz and Priller, 2014; Prinz et al., 2014).

Later, in 1928, Río-Hortega was appointed head of biological research at the Institute of Cancer in Madrid. From there he continued to work on microglia and oligodendrocytes, among other topics comprising the morphology and function of intracellular organelles; the classification and nomenclature of brain tumors; and the histology of the pineal gland, the hematopoietic organs (spleen), the digestive and urinary systems, etc. (Cano Diaz, 1985). Due to his leftish political views, and in spite of having a worldwide recognition (he was twice nominated for the Nobel Prize), he had to leave Spain during the Civil War (1936–1939). He spent some time at the Hôpital de la Pitié, Paris and the University of Oxford, UK, where he was awarded an honoris causa doctorate. He then exiled to Argentina in 1940 to settle a new lab and continue his research until he died of a self-diagnosed genital cancer in 1945 (Castellano and Gonzales, 1995; Palmero and Del Río-Hortega, 2002). His legacy continued with Isaac Costero, a student in his lab who learned tissue culture techniques at the Paul Ehrlich Institute in Frankfurt, Germany and implemented the first culture of microglia from the human brain, which he imaged in time-lapse to confirm the motility and phagocytic capacity of microglia (Rezaie and Hanisch, 2014); and with Wilder Penfield, who became a famous neurosurgeon specialized in the treatment of epilepsy, notorious for his functional mapping of the brain, and a pioneer in the study of glial scars (Gill and Binder, 2007).

## The renaissance of functional neuroanatomy

**Figure 2:**
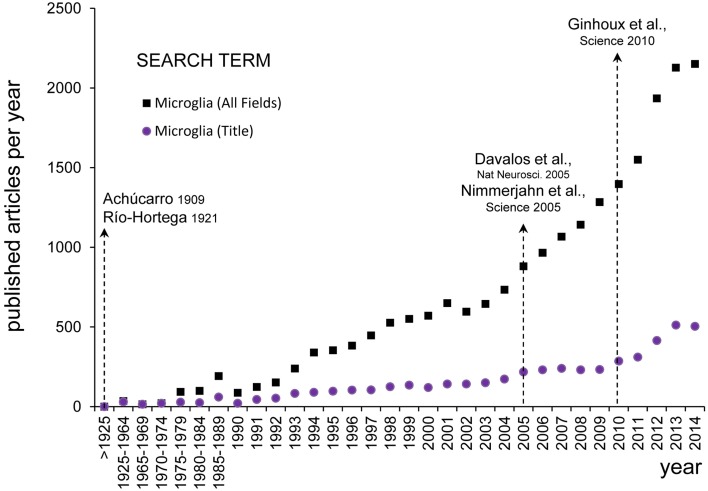
**Evolution of microglial research**. The numbers of published papers per year about microglia was assessed on PubMed Central (www.ncbi.nlm.nih.gov) using the search term “microglia” (All Fields) and setting the Date of Publication from January 1st to December 31st from 1900 until 2014 (black squares). Since the earliest descriptions of Achúcarro and Río-Hortega at the beginning of the twentieth century, there were barely any papers discussing microglia published before 1990 (456 papers in total), from when there was a steady increase until the end of the Century (356 papers per year). It was not until the early 2000s that there was an exponential growth of microglial research (1180 papers per year in 2000–2014). The inflexion point seems to be located around 2005, after the publication of the seminal papers by Davalos and Nimmerjahn on the extraordinary motility of microglia in the adult brain in physiological conditions (Davalos et al., 2005; Nayak et al., 2014). To detect more specifically papers focused on microglia, we performed another search looking for the term “microglia” in the title (Title Field) (purple dots). Over the period 1990–2014, the percentage of papers with “microglia” in their title over the total number of papers including “microglia” in any field was maintained at a fairly constant rate of 24 ± 0.9% (mean ± SEM). When searching for papers with “microglia” in their title a second inflexion point is also evident around 2010 (155 papers per year in 2000–2004, 231 papers per year in 2005–2009, and 406 papers per year in 2010–2014), coincident with the publication of the influential paper by Ginhoux on the unique origin of microglia from the embryonic yolk sac (Ginhoux et al., 2010). This figure does not intend to be a systematic analysis of the most influential papers on microglia research, but to evidence that the rediscovery of the findings by Achucarro and Río-Hortega shapes our current research in microglial biology.

The principles of functional neuroanatomy established by the Cajal school of thought are now more than ever in vogue in microglial biology: a detailed and systematic morphological analysis of the various cell types and their interactions one with another, that leads to biologically relevant functional hypotheses which can then be directly tested using tools from modern molecular biology combined with state-of-the-art imaging. To this renaissance illustrated in Figure 2, three imaging techniques have been critical in the last decade: transmission electron microscopy (TEM) to obtain high resolution images; confocal microscopy to scan large areas of tissue; and two-photon microscopy to observe microglia in real time in the living brain. In particular, three postulates derived from the original observations of Achúcarro and Río-Hortega, on which the second part of our review focuses, still shape our current research on microglia: (1) their unique origin, (2) their morphological plasticity, and (3) their extreme capacity for phagocytosis [for a systematic review of different aspects of the history of microglial research refer to (Rezaie and Hanisch, 2014)].

A major revolution in the field came from studies showing that, unlike most tissue resident macrophages, microglia are not constantly replaced by bone-marrow derived monocytes from the blood, but in fact are seeded during early embryonic development from the infiltration of yolk sac derived precursors (Ginhoux et al., 2010; Schulz et al., 2012; Kierdorf et al., 2013). The ectodermal vs. mesodermal origin of microglia was a contended topic at the times of Achúcarro, Río-Hortega, and Cajal, and still continued to be highly debated until the 1990s, when it finally became clear that microglia belong to the myeloid lineage (Ginhoux et al., 2013). This idea settled the field for over two decades, during which mature microglia were mostly considered as macrophages orchestrating the brain inflammatory response to pathological insults, even though these cells were also shown to have a remarkable down-regulated inflammatory phenotype in the healthy brain, and to be extremely stable and long-lived in comparison with other macrophage populations (Lawson et al., 1992). In fact, it was not until their precursors from the yolk sac were traced using transgenic fate mapping strategies (Ginhoux et al., 2010; Schulz et al., 2012; Kierdorf et al., 2013) that the community realized their unique properties and functions, particularly in normal physiological conditions. Their repertoire of physiological roles discovered so far in the developing and mature CNS comprises the regulation of neural progenitors survival, blood vessel growth, developmental cell death, axonal sprouting, and neuronal firing, synaptic activity and plasticity, among others, in addition to neuronal circuit remodeling through the phagocytosis of newborn cells and synaptic elements as discussed below (Eyo and Dailey, 2013; Bilimoria and Stevens, 2014; Nayak et al., 2014).

Another breakthrough occurred in the last decade when microglial dynamics were examined for the first time in the intact brain of living animals, through the skull of transgenic mice where they were fluorescently labeled, using two-photon microscopy (Davalos et al., 2005; Nimmerjahn et al., 2005). These important observations confirmed Achúcarro and Río-Hortega's hypothesis that microglia are extremely dynamic (Achúcarro, 1909; Río-Hortega, 1919b). The degree of their structural plasticity surpassed all expectations: microglial processes were found to survey their surrounding environment on a time scale of minutes, in contrast to neurons and other types of neuroglial cells which show no comparable structural remodeling *in vivo* (Majewska and Sur, 2003; Eom et al., 2011; Hughes et al., 2013; Lamantia et al., 2014). With these observations, microglia have emerged as the most dynamic cell type of the mature CNS. Their dynamism is far from being random, but microglia continuously respond to neuronal activity and behavioral experience in the healthy brain (Davalos et al., 2005; Nimmerjahn et al., 2005; Wake et al., 2009; Tremblay et al., 2010). From the recent literature, an important distinction between microglial motility (of processes) and mobility (migration of cell body) has to be made (Eyo and Dailey, 2013). In particular, microglia are extremely motile in the mature healthy brain, even though they migrate very little (Tremblay et al., 2010; Hefendehl et al., 2014). During normal aging and neurodegenerative conditions, including Alzheimer's disease and amyotrophic lateral sclerosis, however, microglia acquire the ability to migrate, confirming Río-Hortega's idea that it is during pathological conditions that microglia would show their dynamic behavior at best (Río-Hortega, 1919b). In these conditions, their surveillance of the environment (motility) is concomitantly reduced (Damani et al., 2011; Dibaj et al., 2011; Krabbe et al., 2013; Hefendehl et al., 2014), possibly because they become involved with many other tasks that are required for the inflammatory response.

Microglial motility is not fully understood but has emerged as a major property underlying their immune surveillance function during normal physiological conditions. It has been estimated that microglia could scan the whole brain parenchyma every few hours (Davalos et al., 2005; Nimmerjahn et al., 2005; Wake et al., 2009; Tremblay et al., 2010). Among the structures contacted by microglia in the healthy brain stand out synaptic elements, particularly pre-synaptic axon terminals and post-synaptic dendritic spines. Microglial contacts with synaptic elements have been observed *in vivo* with two-photon microscopy, showing durations varying between 5 and 30 min, as well as in fixed tissue using TEM (Wake et al., 2009; Tremblay et al., 2010; Sogn et al., 2013). Importantly, these contacts are widespread, because almost all (~94%) microglial processes directly juxtapose synaptic elements, axon terminals, dendritic spines, perisynaptic astrocytic processes, and synaptic clefts in decreasing order of frequency, in adolescent mouse cerebral cortex (Tremblay et al., 2010). Serial section TEM with 3D reconstruction further revealed that a single microglial process can make multiples contacts with synaptic elements, at multiple synapses simultaneously, sometimes with morphological specializations in the form of finger-like protrusions wrapping around dendritic spines and axon terminals (Tremblay et al., 2010). Clathrin-coated vesicles, which are responsible for the endocytosis of membrane-bound receptors and their ligands (Le Roy and Wrana, 2005), were also observed inside of microglial processes and synaptic elements, specifically at their sites of contact (Tremblay et al., 2010), suggesting reciprocal exchange of molecular signals (which remain to be identified) via clathrin-mediated endocytosis. These two types of specializations indicate that never-resting microglia interact functionally with excitatory synapses (Tremblay and Majewska, 2011). While the function of these contacts remains to be determined, it is now clear that in normal physiological conditions microglia both sense and react to neuronal activity (Miyamoto et al., 2013; Wake et al., 2013; Tremblay et al., 2014).

The last seminal observation of Achúcarro and Río-Hortega concerned microglial capacity for phagocytosis. Microglia are indeed the brain professional phagocytes, and they engulf cellular debris in larger amounts and more rapidly than other brain cells with phagocytic capacity, such as astrocytes (Parnaik et al., 2000; Magnus et al., 2002). A longstanding view over the last century is that microglia need to be activated in order to become efficient phagocytes, and that phagocytic microglia should necessarily adopt an ameboid morphology (Sierra et al., 2013). However, recent findings show that ramified, surveillant microglia are true phagocytes that engulf cells undergoing apoptosis, the major form of cell death, through terminal or *en passant* branches in the adult healthy brain (Sierra et al., 2010). Using as a model the hippocampal neurogenic cascade, where newborn neurons undergo apoptosis throughout adulthood, and a combination of TEM, confocal microscopy, and unbiased stereology methods of quantification it was shown that apoptosis and microglial phagocytosis are tightly coupled, and that phagocytosis of apoptotic cells is fully executed under 90 min on average (Sierra et al., 2010). Two-photon *in vivo* imaging and TEM also revealed that microglia make use of their potential as phagocytes in the healthy brain to eliminate axon terminals, dendritic spines, and possibly entire excitatory synapses during post-natal development, adulthood and aging. In particular, microglial processes harboring phagocytic structures were encountered *in vivo* (Nimmerjahn et al., 2005; Tremblay et al., 2010). At the ultrastructural level, phagocytic inclusions with distinctive features of axon terminals (synaptic vesicles) and dendritic spines (post-synaptic densities) were frequently observed inside of microglial cell bodies and processes, and their prevalence was found to be regulated by neuronal activity and behavioral experience (Tremblay et al., 2010, 2012; Paolicelli et al., 2011; Schafer et al., 2012). This unique capacity of microglia to engulf and remove cellular debris is undisputable, but many open questions remain: Do microglia actively select which cells or neurites/spines/synapses require to be removed? What are the downstream effects of phagocytosis on microglia, synapses, and the surrounding brain parenchyma? What are the functional consequences on neuronal plasticity, learning and memory? Is microglial phagocytosis in the diseased brain equally fast and efficient, and how does it contribute to disease pathogenesis?

## The future of microglia

In these 100 years of microglial history, functional neuroanatomy based on different microscopy techniques has played a central role in enabling us to study these cells in their normal physiological state, thus providing invaluable insights into their possible implication in the pathogenesis of various neurodevelopmental and neurodegenerative diseases where changes in their morphology, dynamics, and gene expression pattern have been described. A recurrent problem across the years has been the development of selective staining tools to specifically visualize microglia and manipulate their gene expression, as required to investigate their causal relationship to the pathogenesis of diseases. To date, and to the best of our knowledge, all “microglia-specific” transgenic mouse lines expressing fluorescent reporters such as the green fluorescent protein (GFP) are not microglia-specific, as they also label the small proportion of brain macrophages located in the meninges and perivascular spaces of the brain (Davalos and Fuhrmann, 2014), as well as the peripheral, circulating monocytes which could contribute to replenishing the microglial population under certain (Gomez Perdiguero et al., 2015) pathological conditions (Ginhoux et al., 2013). Furthermore, the most commonly used reporter mouse line for live imaging and inducible expression based on the Cre recombinase system is a knock-in where the constructs replace the endogenous fractalkine receptor (CX3CR1) locus (Goldmann et al., 2013; Wolf et al., 2013; Yona et al., 2013). Although the heterozygous CX3CR1^GFP/+^ mice have been extensively used for imaging, they show functional deficits in synaptic plasticity, learning, and memory compared to wild-type mice (Maggi et al., 2011; Rogers et al., 2011) and thus may not be the best model to study the roles of microglia in the healthy brain.

In recent years, fate mapping strategies have enabled to specifically visualize cells of the microglial lineage (Ginhoux et al., 2010; Schulz et al., 2012; Kierdorf et al., 2013). Last year, gene profiling and quantitative mass spectrometry analysis also allowed to identify for the first time a molecular “signature” of microglia in the healthy brain [which was subsequently shown to be altered in neurodegenerative diseases (Butovsky et al., 2014a)], leading to the development of selective antibodies to visualize particular phenotypes of microglia vs. monocyte-derived macrophages (Butovsky et al., 2014b). Novel microglial-specific fluorescent probes such as CDr10a and CDr10b may also be useful for live imaging (and isolation), although their specificity remains to be tested (Leong et al., 2014). In the near future, these advancements should lead to the development of improved strategies for both the visualization and conditional manipulation of gene expression in microglial cells of various phenotypes, leading to an explosion of discoveries regarding their distinctive roles across brain development, function, plasticity, and disease.

### Conflict of interest statement

The authors declare that the research was conducted in the absence of any commercial or financial relationships that could be construed as a potential conflict of interest.
